# Correction to “Targeting T Cell–Mediated Inflammation: Clinical Benefits of Lifitegrast in Improving Symptoms and Signs of Dry Eye Disease”

**DOI:** 10.1155/joph/9857150

**Published:** 2026-11-05

J. Xiaodan, L. Yuanhong, W. Hongmei, W. Yiren and L. Xuemin, “Targeting T Cell–Mediated Inflammation: Clinical Benefits of Lifitegrast in Improving Symptoms and Signs of Dry Eye Disease,” *Journal of Ophthalmology*, 2026, 9973882, https://doi.org/10.1155/joph/9973882.

In the article, an affiliation for Hongmei Wang was erroneously omitted:

• Eye Hospital of Wenzhou Medical University in Wenzhou, Zhejiang Province, China

The corrected author list and affiliations are shown below and have been corrected in the article.

Xiaodan Jiang ^1,2,†^, Yuanhong Li ^1,2,†^, Hongmei Wang ^1,2,3,†^, Yiren Wang ^1,2^, Xuemin Li ^1,2,∗^

1. Department of Ophthalmology, Peking University Third Hospital, Beijing, China

2. Beijing Key Laboratory of Restoration of Damaged Ocular Nerve, Beijing, China

3. Eye Hospital of Wenzhou Medical University in Wenzhou, Zhejiang Province, China

Additionally, the authors wish to correct the article to state that Hongmei Wang shares first authorship and contributed equally with Xiaodan Jiang and Yuanhong Li. The updated author statement is below and has been corrected in the article.

“Jiang Xiaodan, Li Yuanhong and Wang Hongmei have contributed equally to this work and share first authorship.”

We apologize for these errors.

